# Perceptions and actions of healthcare professionals regarding the mother-child relationship with premature babies in an intermediate neonatal intensive care unit: a qualitative study

**DOI:** 10.1186/1471-2393-14-313

**Published:** 2014-09-08

**Authors:** Camila Fleury, Mary A Parpinelli, Maria Y Makuch

**Affiliations:** Department of Obstetrics and Gynecology, School of Medicine, University of Campinas (UNICAMP), Campinas, São Paulo Brazil; Campinas Center for Research in Reproductive Health (CEMICAMP), Campinas, São Paulo Brazil

**Keywords:** Mother-child relationship, Holding, Intermediate care nursery, Healthcare team, Qualitative study

## Abstract

**Background:**

The mother-child interaction after delivery may be hampered when the newborn baby is hospitalized. The objective of the study was to understand perceptions and actions of healthcare professionals (HCPs), working in an intermediate neonatal intensive care unit (NICU), regarding mother-child relationship of hospitalized premature babies in the first weeks after delivery and the professionals’ support for the development of this relationship within an NICU environment. The psychoanalytic concept of *holding* defined by Winnicott was used as the theoretical framework.

**Methods:**

A qualitative study was conducted with 20 HCPs (physicians, medical residents, nurses, and nurse technicians) working at an intermediate NICU of a referral hospital in Brazil. Semi-structured interviews were conducted, recorded and transcribed verbatim; and thematic analysis was performed.

**Results:**

The HCPs referred to the difficulty that these mothers had to develop the mother-child relationship within this environment. If they observed that the mother had initial inhibitions to interact with her baby, they tried to facilitate this process, since they were aware of the importance of early bonding for the child’s well-being. They attributed the mothers’ difficulty to the fragile appearance of the premature baby, the limited contact often imposed by the routine of the unit and the lack of participation in the decisions regarding the care given to her baby. HCPs tried to help women bond with her child by giving support and encouragement. Most of the physicians reported that the nurses represented a link between physicians and the mothers of the hospitalized babies.

**Conclusion:**

The HCPs reported attitudes and actions indicative of *holding.* A more in-depth understanding of the relationship between HCPs and mothers of premature babies at an NICU during the first days after delivery, and the needs of the mothers and her baby to be close to facilitate bonding should be part of the routine discussions of the NICU health team.

## Background

After premature childbirth evidence highlights the benefits of early relationship and bonding between a mother and her newborn infant when the baby is still hospitalized. Physical contact, understanding and caring for the baby’s needs and emotional bonding between the mother and her premature baby is essential for the development of the newborn and should be encouraged by healthcare professionals (HCPs) working at intensive care units for these babies. All actions taken in this direction should take into account cultural variations [1–3].

A premature birth may alter women’s expectations with respect to their role as a mother. Women often feel incapable of caring for, or protecting her baby, and this feeling interferes with the way they interact with the newborn and the development of the mother-child relationship [4, 5]. The support of HCPs may help mothers of premature babies to deal with the difficulties associated with this situation, to seek and establish closeness and bonds of attachment with their babies [6–8].

Recent studies evidence the importance of the care, support and the relationship established between nurses, the newborn infant and their parents, both mothers and fathers, in the environment of neonatal care units [7, 9, 10]. A good relationship between the parents and the nursing staff is fundamental to enable parents to remain close to their babies during the time they are hospitalized [11, 12]. Despite recognition of the importance of the parents’ proximity for the infant’s recovery, HCPs working at these units report that interaction with the family of the hospitalized child is one of the difficult aspects of their work [13]. Furthermore, these HCPs were aware of the difficulties involved in taking on the role of facilitators of the development of the early relationship between parents and their infants [14, 15]. There are reports that refer that HCPs often provide instrumental support and technical care that, albeit necessary and indeed vital for the babies’ recovery, does not focus on the desires and needs of the parents, frequently marginalizing their role during the hospitalization period [5, 16, 17].

Information on the perspectives of HCPs regarding the support given to mothers of hospitalized babies for the development of the mother-child relationship and on the role they could play to facilitate the development of this relationship in a hospital environment is scarce. The objective of this study was to understand the perception and actions of HCPs with regard to the mother-child relationship in the first weeks after birth within the setting of an intermediate neonatal intensive care unit (NICU) and the support the HCPs give mother’s for the development of this relationship.

## Methods

### Theoretical framework

The theoretical concept of *holding* defined by Donald Winnicott [18], a pediatrician and psychoanalyst, was the conceptual tool. This concept was used to evaluate the perceptions and attitudes of the HCPs of an intermediate NICU with respect to the development of the mother-child relationship within this hospital environment and the support they gave for the development of this relationship. The term *holding* originates from the verb “to hold” in the sense of giving support or establishing favorable conditions for development. Winnicott initially used this concept to describe the importance of the physical and psychological support mothers gave to their babies for their initial development.

Later, the author expanded the use of this concept to characterize the work of HCPs whose basic function is to provide care [19]. Within this conception, the attitudes and actions of HCPs when caring for their patients could be considered similar to the care, the understanding of needs, and support mothers give their babies. This support (holding) creates favorable environment for development. In the case of HCPs, according to this theoretical concept, attitudes related to caring, human understanding and love creates a favorable environment for recovery and healing. *Holding*, therefore, refers to the sensitivity of HCPs to perceive the patient’s needs and respond focused on the needs of an individual in a certain situation [20].

Winnicott emphasized women’s need for *holding* in the postpartum period, affirming the importance of adequate communication, good relationship and understanding between the physicians and nursing staff and women in the post-partum period. During post-partum, in general, women tend to be physically exhausted and psychologically fragile, depending on the healthcare team in many different and everyday circumstances [19]. If they are unable to count on efficient professional support, serious difficulties may interfere with the development of the mother’s capacity to bond with her baby [21], principally in the case of mothers who need to develop this relationship within the setting of a neonatal care unit.

### Type of study

A qualitative study was conducted with the objective of gaining an in-depth understanding of the perspectives and attitudes of HCPs working in an intermediate NICU on the development of the mother-child relationship of mothers with premature babies within a hospital environment. Semi-structured interviews were used in the present study to understand the perspective of the HCPs of an NICU, their beliefs, actions and responses to this particular health issue. Also, to understand the support *(holding)* they provided to mothers and babies under their care at an intermediate NICU for the development of the mother-child relationship. This approach permitted to hear the stories these professionals had to tell regarding to their everyday experience of caring for premature babies and relating to the babies mothers; and to explore the meanings this experience had for them [22, 23].

The study was conducted at the intermediate NICU of a tertiary referral hospital for high-risk pregnancy in the southeastern region of Brazil. The hospital is part of the Baby-Friendly Hospital Initiative (BFHI) launched in 1990 by the World Health Organization (WHO) and the United Nations Children’s Fund (UNICEF) to promote breastfeeding, rooming-in and encouraging mothers to be the baby 24 hours a day [24].

The definition adopted in this study for NICU was a nursery that has the capability of providing intermediate neonatal care services for neonates and infants who do not require intensive care but require care at a level higher than provided in a general nursery. To receive care babies are separated from their mothers. The Ethical Committee approved the study protocol and all participants signed an informed consent form.

### Recruitment and sampling

Participants were selected according to purposeful sampling criteria and the strategy was maximum variation sampling [23, 24]. Participants were selected according to a common characteristic: HCPs providing care at an intermediate NICU and the variation was in the different roles and functions of the professional within the healthcare unit. The number of participants was determined following the criterion of data saturation [25].

Twenty HCPs (physicians, third-year medical residents, nurses, and nurse technicians) were interviewed. They were provided with information on the study objectives and on the voluntary nature of participation during a routine monthly administrative meeting or through personal contact with one of the investigators. A standardized form was used to obtain information on the socio-demographic characteristics and general data regarding the professional activities of the participants.

The interviews were held in Portuguese, in private, by appointment and lasted approximately one hour, between February and October 2012. All were conducted by one of the investigators (CF), a psychologist, who was not a member of the hospital staff. Before initiating the interview, participants were asked about their experience of working in the area of caring for premature babies. The interview guide included topics related to the participants’ experience working in an intermediate NICU, their perspective on the development of the mother-child relationship and their perception on the support (*holding*) they provided in their interactions with the mothers of premature hospitalized babies. The interview topic guide contain the following items:

1. Talk to me about your experience of working in neonatology. In your opinion, what are the most important aspects that characterize your daily work? What is your greatest challenge at work?
2. In your opinion, what is it like for the mothers whose babies are in hospital?
3. How is the contact between these mothers and their babies? Do they try to get close to their babies and give them attention or do they not? Why?
4. How do you think the mothers feel when they take care of their baby?
5. How is your contact with the mothers during your work?
6. When you receive a mother at her first visit to the intermediate care nursery, how do you receive her? What information do you give her? Do you think this information is sufficient or not? Why?
7. Do you try to establish a bond between the mother and child or not? If yes, how? When the mother is finding it difficult to touch/care for her baby, do you do anything or not?
8. What do you believe facilitates contact between the mother and her child during hospitalization? Do you believe that a certain attitude from the healthcare team could interfere negatively in how the mother interacts with her child or cares for it?

### Data analysis

Interviews were transcribed verbatim and transcripts checked for accuracy against the recordings [23, 24]. An initial thematic frame was organized based on topics of the theoretical concept of *holding.* Relevant themes regarding the perception and attitudes of the HCPs on the development of the mother-child relationship within the intermediate NICU environment, their role in supporting and encouraging the development of this relationship were grouped. During the initial phase of analysis, while reading through the interviews, the salient topics and the recurring perceptions, attitudes, and experience of the HCPs were organized in a meaningful way.

**Figure 1:**
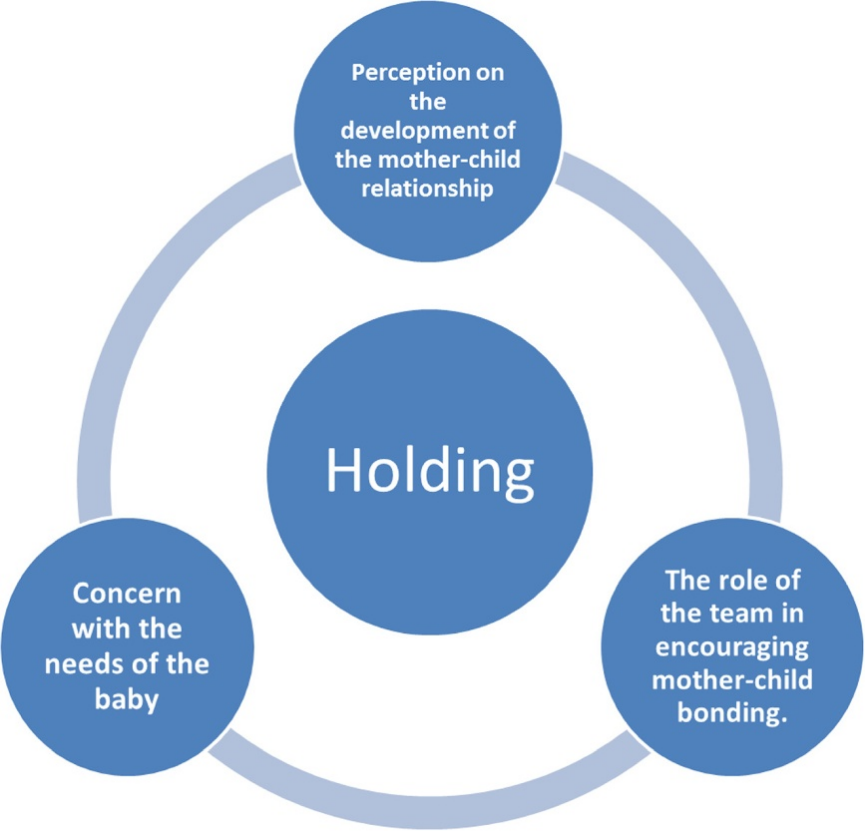
**Categories of analysis.**

The main themes identified that were considered relevant to understand the perceptions and attitudes of the healthcare team were: their perception on the development of the mother-child relationship, their concern with the infant’s needs, the support needed by the mothers and their role as caregivers in offering support and facilitating the development of this relationship (Figure 1). Subsequently the information coded in the interviews was confronted with relevant aspects of what, according to the theoretical framework adopted, is considered *holding.* At this stage of analysis the themes and the illustrative examples of what participants said were organized by one of the authors a psychologist (CF), and in parallel this same procedure was performed by another author (MYM) a clinical psychologist with experience in working with the theoretical framework of Winnicott in clinical and research settings. Data was confronted, issues were discussed and only the results obtained after consensus were included. The quotes used as illustrative examples of what participants said were translated from Portuguese to English, and back translated into Portuguese by one of the authors (MYM) who is fluent in both languages.

### Details of ethical approval

This study was approved by the Ethical Committee of the School of Medicine, State University of Campinas (UNICAMP), under registration number 1202/2011.

## Results

Twenty HCPs working different shifts at the intermediate NICU were interviewed (Table 1). Half the HCPs interviewed had worked at the unit for at least six years. All the physicians worked 24-hour shifts, while the nursing staff was divided between day and nightshift workers. Most of the participants were female, since the staff at intermediate NICU where the study was conducted was predominantly female.

**Table 1:** **Characteristics of the study participants**

Characteristics	Participants (n = 20) N
***Profession***	
Assistant physician	5
Medical resident	5
Nurse	5
Nurse technicians	5
***Sex***	
Female	16
Male	4
***Age (years)***	
26-31	7
32-37	3
38-43	1
44-49	1
50-55	6
≥ 58	2
***Time of service (years)***	
≥ 1	3
1-5	5
6-11	2
12-17	5
18-23	3
≥ 25	2
***Work shift***	
***Medical team***	
24-hour shifts	10
***Nursing team***	
Day shift	6
Night shift	4

### Perception of the development of the mother-child relationship

Physicians and nurses referred to the difficulty mothers of babies cared for in an intermediate NICU had to develop the mother-child relationship within this environment. They observed that for most mothers it was a difficult experience to be separated from her baby immediately after giving birth. At the intermediate NICU mother’s initially seemed insecure and waited for permission from the healthcare team to be close, touch or care for her baby even when they had received authorization to do so. They attributed the mothers’ difficulty to the fragile appearance of the premature baby, the limited contact often imposed by the routine of the unit and their lack of participation in the decisions regarding the care given to her baby.

*“…there’s the mother who at the beginning doesn’t even go up to the incubator or the crib, if the baby is in a crib…but it’s not because she doesn’t like the baby or for any lack of love, it’s nothing like that, it’s actually fear, insecurity* (nurse technician)

According to physicians and nurses, during first contacts with the baby in the intermediate NICU, many women expressed feelings of guilt for having had a baby that was not well and needed hospitalization. They said that these women questioned themselves whether they had done something during pregnancy that could have harmed the baby or if their body was not good enough to gestate a healthy baby.

*“So I think that to greater or lesser extent these mothers feel that something was lacking in them, that they are not as good for their baby as other mothers”* (physician)

Most of the HCPs, independently of their functions, reported having observed that, when the mothers initiated contact with their babies in the intermediate NICU environment most of them were very concerned with the baby’s situation and some frightened to approach them. They felt a great need to touch and hold her baby and when they were able to do so they became calmer. These HCPs also perceived that as time went by and mothers spend more time with her baby, most of them began to identify themselves as the mother of her baby, became more confident with her ability to care for her baby and started to take the initiative to interact with her baby.

*“…they gradually feel more at ease, become more participative…You can see how they gain confidence as each day goes by…I think even when they can touch the baby, even if they can’t pick it up…just by touching it, they feel in contact with the baby and it calms them…They feel that the baby is real, they touch its hand, they actually allow themselves to believe that the baby is theirs, the child is theirs, not just a baby undergoing treatment…”* (physician)

All the participants reported that the majority of the mothers were very affectionate with their newborn bay. Tried to stay as long as possible with her child, even when still recovering from delivery or when the baby needed treatment or interventions that limited direct contact. The mothers talked to their babies, caressed them and some sang to them. Some mothers, when unable to be present, maintained telephone contact.

*“…most of the time, they try to give as much of themselves as they can to their babies, irrespective of how they themselves are, because many are still recovering from delivery…and a bit unsteady…in pain…sometimes still weak…and they go to a great effort to be present…most of the mothers…are very affectionate, they want to touch their baby…Some even rock the baby in the incubator with their hands, moving it, you know, by rocking its bottom to calm it in the incubator…”* (nurse)

### Concern with the baby’s needs

The majority of the HCPs referred to the importance of contact between the mother and her baby, to the baby’s need to be close to the mother and to receive her care. They believed that the feelings, the closeness and the presence of the mother contributed to the baby’s well-being and recovery and the production of breast milk. Because of these convictions, whenever they noticed that a mother was not present, they tried to find out why and to identify ways of helping and encouraging her to be present.

*“So I think we always expect the mother to bond, to help her develop a good relationship…in this case we were worried…all right, so the father was doing everything right….but we weren’t happy; we wanted the mother to be here, and then she came and we were happy, she was able to take care of her baby and to take it home…”* (physician)

*“It seems to go round in a circle, right? I visit my baby more often, I pick it up and my bond with it grows, I have more milk, so when the time comes for the baby to feed from my breast, it’ll be easier. It seems that … this relationship develops better and it even contributes to allowing the baby to leave hospital faster”* (nurse)

Nurses reported that their priority was the well-being of the babies in the intermediate NICU and that they made efforts to identify any possible problems or discomfort they may have, paying attention to all the details of the care provided. Furthermore, some nurses considered that this focus on the baby’s well-being sometimes led them to forget about the mother’s needs and to limit possibilities of care, proximity and direct contact of the mother with her baby.

*“First the well-being of the baby….Sometimes it’s restless because something is wrong; sometimes it’s just the way the baby is, you have to know that … You need to know what is going on, what is going on with the baby …but you need to see it from the mother’s point of view too. She’s tired…because sometimes they tell her that she has to breastfeed, she has to breastfeed…So sometimes we…we are very focused on the baby and demanding things of her…”* (nurse)

Most of the interviewed physicians expressed concerns with the mother’s ability to care for her premature baby, since these babies behave differently from a full-term infant; they are sleepier and less responsive. They believed that mothers would find it difficult to understand and identify the needs of premature babies and that they needed to receive professional and specialized guidance.

*“They think…that since they are mothers they will know what the baby needs, you know?…that it will be instinctive and she’ll know when she’s home what she has to do to ensure that the baby is all right…most of the time, with babies born at term, that’s fine, but with premature babies it’s not. The behavior of premature babies is not like that of the other children…”* (physician)

Some HCPs, particularly the nurse technicians, reported that concern with the needs of the premature baby ended up with them assuming the responsibility for certain tasks that should have been performed by the mothers. They also recognized that sometimes it is difficult for the mother to hold her baby or to perform some tasks of caring that could facilitate the mother child relationship because the HCPs of the NICU believe that those attitudes could hamper the routine care provided to the baby.

*“What often happens is that we’ve just finished taking care of the baby, got it all settled down, so we make decisions such as: no, you’re not going to pick the baby up now that it’s sleeping, we need to let a premature baby sleep … we’re afraid, we have our doubts, it’s a concern of ours… letting this mother take charge of this baby…”* (nurse technician)

### The role of the healthcare team in mother-child bonding

The majority of the participants reported that, in general, the HCPs tried to help women bond with their child by giving support and encouragement. Many said that good communication with the mothers was important, supported and helped them in the process of bonding with their babies. Gestures and words had to be used with care to ensure that their own knowledge was not imposed upon the mothers, since that would interfere negatively in the bonding process.

*“Maybe we do interfere…with what the mother knows, and we want to impose our knowledge on her, right, but I think this has a negative effect, because she may think that what she believes to be right, isn’t right….so what is right? This might make her insecure with respect to caring for the baby”.* (nurse)

The support given to the mothers by the doctors was different from that given by the nurses. The doctors gave support, mainly, by providing mothers with clinical information on the baby’s condition. Before giving them the diagnosis, they evaluated each mother’s level of knowledge about the child’s status and transmitted the information clearly and carefully, since, as they had already observed, failure to understand the diagnosis would induce feelings of guilt or uncertainty in the mothers.

*“…we’re very careful when we tell them things, right, sometimes we, it’s…we might make a comment that the mother may not understand…it might frighten her, she might get worried, insecure, I think this could be bad, yes…they don’t know to what extent it was something they did that might be causing whatever is wrong with the child, right…and sometimes we might say, oh, it was that infection inside the uterus or it was the mother’s high blood pressure, so we end up blaming the mother, unintentionally”.* (resident physician)

All the resident physicians reported concern with the type of contact that they established with the mothers. They questioned whether the type of care provided encouraged the mother-child bonding process or not and whether it was sufficient or if they should be doing something more to help.

*“…it’s a shortcoming that I feel I need to correct. I don’t normally seek contact with the mothers, I…my contact with them is more in the sense of responding to demand, for example, the mother wants to talk to me, so the nursing staff tells me ‘that mother is here and wants to talk to you, can you talk to her?’”* (resident)

The majority of the physicians reported that the nursing staff represented a link between physicians and the mothers of the hospitalized babies; were responsible for establishing more intimate communication, providing care and support. They considered that mothers felt more at ease and talked more freely with nurses, whereas with them the mothers tried to talk about what they believed the doctors wanted to hear, perhaps in the hope of anticipating the child’s discharge from hospital.

*“…There are rarely any mothers who say they don’t want to breastfeed, they want to get the baby out of hospital as quickly as possible because they are tired of being in the unit…So we go, talk to them about the risks, everything we need to tell the family, right…and if they have any questions the nursing staff ends up acting as intermediaries. So I think the nursing staff has greater contact with these mothers, in the care…”* (physician)

The nursing staff said that they explained to the mothers that despite its fragile appearance the premature baby could be touched and held, and tried to encourage them be close to the baby. When they perceived that the mother was having difficulty in establishing contact, they gave support for different ways of interacting with her baby within the hospital environment, emphasizing that, even if it was not possible for her to hold the baby, she could touch the baby, look at the baby and talk to the bay, and that this was good for the baby and for the mother-child bonding process. Some of these HCPs believed that the lack of privacy for the mother to interact with her child could be a problem during this initial contact and they tried to create opportunities for the mother to be alone with her baby whenever the baby’s clinical condition permitted.

*“The mother might be frightened, insecure…so we have to help her overcome this and explain that she won’t hurt the baby if she touches it…that contact with her will be good for the baby, also to hear her voice… then she starts to believe that contact with her is important”.* (nurse technician)

All nurse technicians reported that having informal conversations with the mothers of the babies they cared for at the NICU to understand their misgivings and difficulties in bonding with their baby. Also they explained, in a simple way in order to facilitate understanding, the information the doctors gave them on their baby’s condition.

*“…you sit down with the mother and talk to her, let her say what she needs to say…if you allow her to feel at ease, she’ll talk, “Oh, I don’t want to touch it”. But why don’t you want to touch it? “It’s because I’m frightened I’ll make the baby worse”. No, you can touch it and you’ll be helping it. Then she’ll get closer to the baby”.* (nurse technician)

More than half the interviewed HCPs reported difficulties in relating to the mothers. Most of the physicians expressed surprise at the difficulty many mothers had in understanding why their baby had to be in hospital, the time the baby would have to stay in hospital or in accepting that the baby’s state of health was deteriorating. They believed that this occurred because the women had received scarce information during prenatal care or because the mothers refused to accept the negative part of the child’s diagnosis. Also, the nurses attributed this lack of comprehension to too much information and to the mother’s current fragile state.

*“…they come in, they talk to us….sometimes they come back and ask the same thing to another member of staff…it’s obvious that they want a positive answer…but a premature baby is always a high-risk patient and if we don’t tell them this, then the mother can turn round later and say: but you didn’t tell me that! You said that everything was fine! Because even when we tell them, they only hear what they want to hear…”* (physician)

*“…it’s a lot of information all at once, right? It’s the baby that isn’t going home, that’s here, but why is it here? And there’s that whole story, the childbirth that she just went through, sometimes she’s still in pain too, sometimes she’s not feeling well. So, initially she can’t deal with it all, all the information”.* (nurse)

According to some participants, the women who spent more time with their baby at the hospital commented and criticized the way in which the HCPs did their job, creating discomfort in the team. According to these HCPs, the difficulty in dealing with these mothers was due to a lack of training the health team to deal with the emotional needs of the mothers of the babies they cared for, and this was considered a disrupting factor in communication.

*“…it’s preparing us for this…to understand this mother. What is missing is for us to receive some guidance, for us to know how to deal with this, it’s difficult. I think that sometimes we…label the mother, this mother is a nuisance, but then you don’t ask yourself why she’s being a nuisance, what is going on with her. What’s behind it?”* (nurse)

## Discussion

The HCPs interviewed in this study understood the importance of creating an environment of *holding* to facilitate the development of the initial mother-child relationship. They were sensitive to the needs of the mothers whose babies were at the NICU, provided support and helped them. According to Winnicott [21], immediately following childbirth a woman needs a support network *– holding –* from those who are close to her. They will provide help, understanding, support and security, minimizing her anxieties and enabling her to dedicate time to her newborn infant. When the infant is in hospital, this network then consists of the HCPs who interact with the mothers in the hospital environment.

The HCPs participating in this study reported that they had observed that for some women it was difficult to interact with their baby, to touch or care for them initially due to their insecurity, to the limitations and rules imposed by the hospital and to the fear of hurting the baby. A previous study showed that the HCPs believed that the mothers’ inhibition was generally due to a fear of hurting the baby or causing it discomfort [26].

The benefits of physical contact between a mother and her baby for the clinical stability and development of the newborn infant have been well documented [27–29]. The HCPs in this study reported that, as time went by, the mothers sought physical contact with their baby and this led to an improvement in the newborn infant’s clinical condition, and the mothers then felt less anxious and more certain that they would be able to take care of their child. Although the HCPs interviewed in this study recognized the importance of mother-child bonding, the focus of their work was on treating the baby. Their concern was for the child’s health and the fear that the mother might find it difficult to recognize the needs of a premature baby. This concern could be the reason why the attitudes and actions of some HCPs were contrary to the concept of *holding*, taking over the baby’s care and imposing limitations on the contact between the mother and her child. A similar attitude of the HCPs was observed in a study in which it was reported that in routine NICU care the mother may be neglected and deprived of participating in the care of her baby [30].

The importance of the HCPs’ support for the parents of babies hospitalized in an NICU following delivery has been the subject of several studies [2, 14, 27]. Our results contributed to this discussion showing that the support given to the mothers by the medical staff was different from that given by the nursing staff. The focus of the medical team was on providing information on the baby’s clinical status and on the care required. Clinical information is one of the principal forms of support given by physicians to the mothers of babies hospitalized in an NICU [16, 17].

In this study, the medical team adopted a *holding* attitude by attempting to establish appropriate communication, transmitting the baby’s diagnosis with care to avoid fomenting feelings of guilt or anxiety in the mothers. These HCPs did not act mechanically, limiting their actions to simply passing on information, but, rather, they sought to meet the needs of each individual mother and child [31]. The importance of the HCPs adopting a *holding* attitude was discussed in a study reporting that the mother who gives birth to a sick or premature child faces anguish and a lack of support. The right word and the support of a third person may serve as a shield, creating conditions that will help her at that time in her life [32].

The nursing team taking care of hospitalized infants has been considered a mediator that may facilitate or interfere in the development of the mother-child relationship [6, 13], responsible for carrying out actions aimed at providing practical care [9, 13]; however, there are often difficulties in promoting actions to improve mother-child bonding [14]. With respect to this difficulty, the results of this study showed the nursing staff took certain actions to facilitate the mother-child relationship such as encouraging contact between the mother and her baby, supporting different ways of bringing her closer to the infant, and promoting informal conversations that allowed the mothers to talk about their difficulties. Allowing the mother to care for her baby and facilitating contact constituted attitudes of *holding.* Winnicott [18, 19] emphasized that the medical and nursing staff should make it possible for the mother to exercise her maternal role within the hospital environment, allowing her to do what she is able to do and avoiding unnecessary interference in the natural processes of the mother-child relationship.

Some HCPs in our study reported difficulties in interacting with the mothers. Similar difficulties have been reported in other studies conducted in NICUs [13, 15, 33], indicating that the continuous interaction between the nursing staff and the parents generates an emotional involvement that hampers the interaction between them. The participants of this study also attributed the difficulty in interacting to the lack of preparation of the healthcare team to deal with the emotional issues of the mothers of hospitalized babies.

A possible limitation of this study was that it was conducted with HCP from only one NICU. On the other hand, the main strength was that the research was conducted within a theoretical framework that allows an in depth understanding of the situation and may indicate possible actions. The results of the present study can be used to discuss strategies to train and give support to HCP, both those who are initiating activities or those already working at NICU, to prepare an adequate setting for mothers and babies do develop the mother-child relationship.

## Conclusion

The HCPs who participated in this study recognized the importance of the development of the mother-child relationship during the time in which the infant is in hospital and, although there were some difficulties in the relationship between the team and the mothers, they sought to support and encourage bonding. The HCPs reported attitudes and actions indicative of *holding*, in accordance with the theoretical reference used in this study. This study may help to clarify certain aspects of the perception of HCPs on mother-child bonding and of the relationship between mothers and healthcare teams that would encourage the development of *holding* attitudes by HCPs. A more in-depth understanding of the relationship between HCPs and mothers of premature babies at an NICU during the first days after delivery, of the needs of the mother and her baby to be close and strategies to facilitate bonding should become part of the everyday discussions of NICU health team.
